# Effect of Grain Size on the Microstructure and Strain Hardening Behavior of Solution Heat-Treated Low-C High-Mn Steel

**DOI:** 10.3390/ma13071489

**Published:** 2020-03-25

**Authors:** Marek Opiela, Gabriela Fojt-Dymara, Adam Grajcar, Wojciech Borek

**Affiliations:** 1 Department of Engineering Materials and Biomaterials, Silesian University of Technology, 18A Konarskiego Street, 44-100 Gliwice, Poland; Marek.Opiela@polsl.pl (M.O.); Wojciech.Borek@polsl.pl (W.B.); 2 Department of Engineering Processes Automation and Integrated Manufacturing Systems, Silesian University of Technology, 18A Konarskiego Street, 44-100 Gliwice, Poland; Gabriela.Fojt-Dymara@polsl.pl

**Keywords:** high-Mn steel, solution heat treatment, fractography, strain hardening, mechanical properties

## Abstract

The low-carbon high-Mn austenitic steel microalloyed with titanium was investigated in this work. The steel was solution heat-treated at different temperatures in a range from 900 to 1200 °C. The aim was to receive a different grain size before the static tensile test performed at room temperature. The samples of different grain sizes showed the different strain hardening behavior and resulting mechanical properties. The size of grain diameter below 19 μm was stable up to 1000 °C. Above this temperature, the very enhanced grain growth took place with the grain diameter higher than 220 μm at 1200 °C. This huge grain size at the highest temperature resulted in the premature failure of the sample showing the lowest strength properties at the same time. Correlations between the grain size, the major strengthening mechanism, and fracture behavior were addressed. The relationships were assessed based on microstructural investigations and fractography tests performed for the deformed samples. The best combination of strength and ductility was found for the samples treated at 1000–1100 °C.

## 1. Introduction

The second generation of advanced high-strength steels (AHSS) effectively combines high strength and ductility as well as formability. The Twinning Induced Plasticity (TWIP) steels belong to a group of high-manganese austenitic alloys but are cheaper when compared to Cr-Ni stainless steels. Their major advantage is the great susceptibility of the austenitic phase on plastic deformation realized through dislocation glide, mechanical twinning, and/or strain-induced martensitic transformation. The group of high-manganese steels includes a wide range of alloys containing from 15% to 30% Mn [1]. Two major chemical composition strategies has been so far elaborated. The first one includes the alloys with a different Mn concentration and 0.5% to 1% C [2]. The function of carbon is to stabilize the γ phase and to harden the solid solution. In the second group, the content of carbon is decreased below 0.1%, whereby there is an addition up to 4% Al and/or 4% Si [3]. The solid solution strengthening caused by the presence of Al and Si compensates the smaller C content and allows one to control the stacking fault energy of the austenite. The low carbon content eliminates the unwanted serrations on stress–strain curves typical for the dynamic strain aging (DSA) phenomenon [4]. The reduced carbon content also improves the steel machinability [1]. Sometimes, the high-Mn steels can be alloyed by chromium or microadditions of Nb, Ti, V, and B. These additions affect the stacking fault energy (SFE) of the alloy and thus a major strengthening mechanism [4,5].

The strain hardening behavior and final mechanical properties of the high-Mn steels depend on structural processes during cold deformation, which are affected by the SFE of the austenitic phase. The SFE is strongly dependent on the temperature and chemical composition [5,6,7]. In general, the stacking fault energy increases with increasing temperature and Al and Cu contents, whereas Cr and Si decrease it. If the SFE is from 12 to 20 mJm^−2^, a partial transformation of the austenite into martensite occurs, taking advantage of the Transformation Induced Plasticity (TRIP) effect [8,9,10,11]. The SFE from 20 to 60 mJm^−2^ favors intense mechanical twinning related to the TWIP effect [12,13,14,15]. At the SFE values higher than ca. 60 mJm^−2^, the partition of dislocations into partial Shockley dislocations is difficult, and therefore the glide of perfect dislocations is the dominant deformation mechanism. A high impact on the dominating deformation mechanism has also been made by the temperature, strain rate, and grain size [14,15,16,17].

The strengthening of the high-manganese steels is significantly related to a grain size. It is assumed that grain refinement to the size of 100 nm < d < 1 µm causes enhanced work strengthening, resulting in a decrease of uniform elongation in tensile tests. Yuan et al. [18] showed that as the grain size increases, yield stress and tensile strength decrease, whereas the elongation values increase. After increasing the grain size from 2.2 to 28.7 µm, the yield stress and tensile strength decreased from 410 and 725 MPa to 232 and 517 MPa, respectively. At the same time, the total elongation increased from 15.4% to 54.2%. The ductility is improved due to the continuous rise in the work hardening rate, which delays necking and the final fracture of the samples [2]. However, a relatively small number of grain boundaries in coarse-grained samples is a reason for the low yield stress. The moderate tensile strength is more dependent on interactions between original grain boundaries and new-formed twin boundaries. Mechanical twins nucleate at the grain boundaries and then easily propagate across the grain interior increasing the elongation in the coarse-grained steels [3,13]. Moreover, Yuan et al. reported [18] that the amount of deformation twins increases with increasing grain size. Deforming the steel of the grain size 2.2 µm brought no deformation twins, whereas they appeared in samples with the grain size ranging from 5.4 to 28.7 µm. As the grain size increased, the deformation twins were formed more easily. Ueji et al. [19,20] confirmed that the reduction in grain size in TWIP steel Fe-31Mn-3Al-3Si (SFE value was 42 mJ/m^2^) caused the strong inhibition of twinning and the significant decrease in plasticity. Lee [21] compared the plastic deformation behavior during stretching Fe-17Mn and Fe-17Mn-0.6C steels and noticed that the reduction in elongation caused by the grain refinement was smaller for the latter.

Dini et al. [22] reported that, during tensile stresses, the number of twins increases with increasing deformation, which means that the strengthening of TWIP steels with high manganese contents increases due to the presence of mechanical twin boundaries, which interact with dislocations (dynamic Hall–Petch effect). The formation of twins gradually reduces the free path of dislocation and leads to the strengthening of the material. Bouaziz et al. [23] found that of all possible deformation mechanisms in TWIP steels, the twins have the most beneficial effect on work strengthening. The increased work hardening rate results in the faster blocking of twinning and dislocation interactions, which is the reason for the lower ductility. The formed mechanical twins divide the austenite grains and reduce the mean free path of dislocations. Therefore, the twin boundaries act as strong barriers to dislocation motion. If the grain size is very small, the work hardening rate is too high in the initial deformation range, whereas it decreases rapidly with increasing strain. Hence, the best combination of strength and ductility occurs for intermediate grain sizes [10,24].

Therefore, the aim of the work is to produce steel samples of different grain sizes through a solutioning heat treatment performed at different temperatures and to assess the effect of grain size on the strain hardening behavior and resulting mechanical properties of low-C high-Mn steel microalloyed with titanium. The aim was to investigate the material response at the high temperature region, which is of primary importance for the industrial practice. The results obtained at 1200 °C are especially important once this temperature is often applied as the initial temperature of hot rough rolling [7]. A lot of published works omit this region due to edge cracks, which are a real problem in high-manganese steels [13]. This is a major novelty of the study.

## 2. Material and Experiments

The tests were carried out on low-carbon high-manganese steel 0.054C-24Mn-3.5Si-1.6Al-Nb-Ti with the chemical composition listed in Table 1. The laboratory ingot of 50 kg was intended to be modified by rare earth elements (Ce and La) to suppress the deformability of non-metallic inclusions.

Samples of the tested steel were subjected to solution annealing at temperatures of 900, 1000, 1100, and 1200 °C. After a 60-min holding time, the samples were quenched in water. The solution annealing of steel in the temperature range from 900 to 1200 °C was intended to produce different grain sizes before static tensile testing. Metallographic tests were performed using light and scanning electron microscopy methods. The preparation of metallographic specimens included classical grinding and mechanical polishing procedures. Samples for the structure observation with the dimensions 20 mm × 15 mm × 4 mm were etched for 30 s in 5% nital. The observations were carried out using a ZEISS Axio Observer Z1m light microscope (Carl Zeiss AG, Jena, Germany). The measurement of the average diameter of austenite grains was carried out in accordance with ASTM E112-10 [25].

In order to examine the effect of the solution annealing temperature on mechanical properties, static tensile tests were carried out at room temperature in accordance with ASTME8/E8M-15 [26]. The tests were performed on the universal ZWICK Z100 testing machine (Zwick Roell, Ulm, Germany), equipped with an extensometer to measure elongation. Flat samples with dimensions of 6.3 mm × 3 mm and a gauge length of 25 mm were stretched at a rate of 0.25 × 10^−3^ s^−1^. Based on the static tensile test, the yield stress, YS_0.2_, ultimate tensile strength, UTS, uniform elongation, UEl, and the reduction in area, RA, were determined. The results were averaged on the basis of three tests performed for each solution annealing temperature. The tests also allowed to determine the work hardening exponent as a function of increasing plastic deformation:

(1) $$n=\frac{d\left(ln\sigma \right)}{d\left(ln\varepsilon \right)}$$

where: *σ*–true stress, *ε*–true strain.

The strengthening exponent n is determined in the range from the deformation value corresponding to the yield stress to the maximum tensile stress value corresponding to the initiation of neck formation in the sample.

The morphological details of the structural constituents and tests of the surface fractures after tensile tests were carried out in the ZEISS high resolution SUPRA 35 scanning electron microscope (Carl Zeiss AG, Jena, Germany) using an accelerating voltage of 15 kV and magnification in the range from 1000× to 30000×. The identification of the chemical composition of the revealed non-metallic inclusions was performed using an EDS energy dispersive spectrometer (EDAX TRIDENT XM4, EDAX, Mahwah, NJ, USA).

## 3. Results

**Figure 1:**
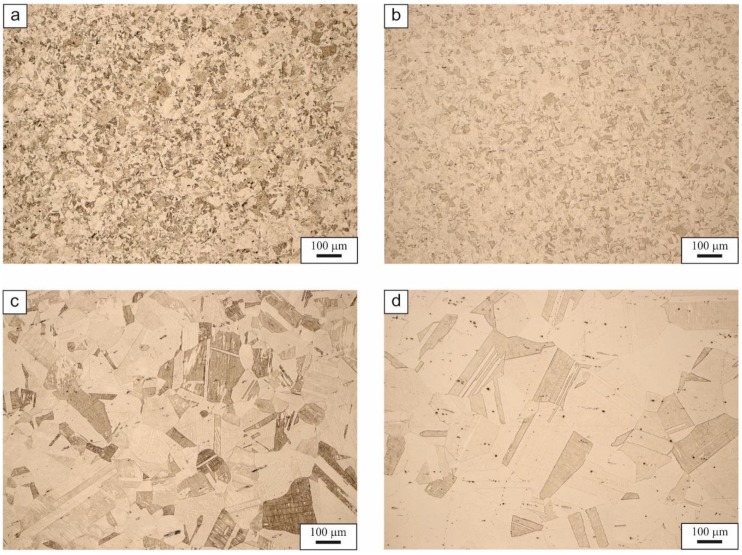
Austenitic microstructures of investigated steel after the solution heat treatment at the temperature of 900 (**a**), 1000 (**b**), 1100 (**c**) and 1200 °C (**d**).

The pure austenitic microstructures of high-manganese 0.054C-24Mn-3.5Si-1.6Al-Nb-Ti steel produced as a result of the solution annealing in the tested temperature range are shown in Figure 1. As can be seen from this figure, the size of austenite grains increases significantly with the increase in solution annealing temperature from 900 to 1200 °C. The intense grain growth is observed after exceeding the solution annealing temperature of 1000 °C. This is a result of the driving force to minimize the energy of the thermodynamic system, which is reflected here as the total boundary energy of all austenite grains. The enhanced grain growth above 1000 °C is due to a gradual dissolution of carbonitrides containing Nb and Ti [4,11].

The samples annealed and cooled in water at 900 and 1000 °C show fine-grained austenitic microstructures with an average grain diameter of ca. 13 and 19 µm, respectively (Figure 1a,b). Increasing the solution annealing temperature to 1100 °C results in a marked increase in grain size and the formation of a coarse-grained austenite microstructure with annealing twins, with an average diameter of 137 µm (Figure 1c). The largest average diameter of austenite grains—ca. 226 µm—is shown by a sample solution annealed at 1200 ° C (Figure 1d). The typical growth of austenite grains after exceeding the solution annealing temperature of 1000 °C is additionally enhanced by the dissolution of Nb-Ti carbonitrides. Detailed data on the effect of solution annealing temperature in a range from 900 to 1200 °C on the size of austenite grains is presented in Table 2.

**Figure 2:**
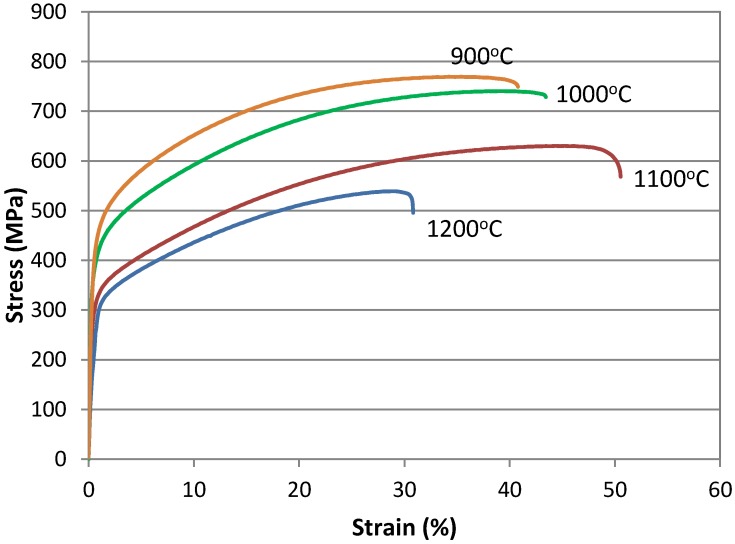
Effect of solution heat treatment temperature on the stress–strain curves registered at different temperatures.

The varied size of austenite grains has a significant impact on the mechanical properties of the steel (Table 3). As expected, the mechanical properties change as the grain size increases. The curves registered during tensile tests at the different temperatures can be seen in Figure 2. One can see that the grain size determined by the annealing temperature has a strong impact both on stress values and ductility. Along with the increase in grain size caused by the increase in the solution annealing temperature from 900 to 1200 °C, the yield stress YS_0.2_ continuously decreases from 480 to 314 MPa, the UTS is reduced from 769 to 539 MPa. However, the corresponding increase in elongation from 39% to 48% takes place only in a temperature range from 900 to 1100°C. The uniform elongation is reduced to 30% at 1200 °C. The ratio of YS_0.2_/UTS ranges from 0.62 to 0.53. The similar tendency of the influence of grain size on mechanical properties was obtained by Ueji and Tsuchida [19,20] in 17Mn-3Al-3Si steel, Yuan and Chen [18] in 25Mn-3Cr-3Al-0.3C steel, and also by Dini [22] in the TWIP steel containing 31% Mn, 3% Al, and 3% Si.

**Figure 3:**
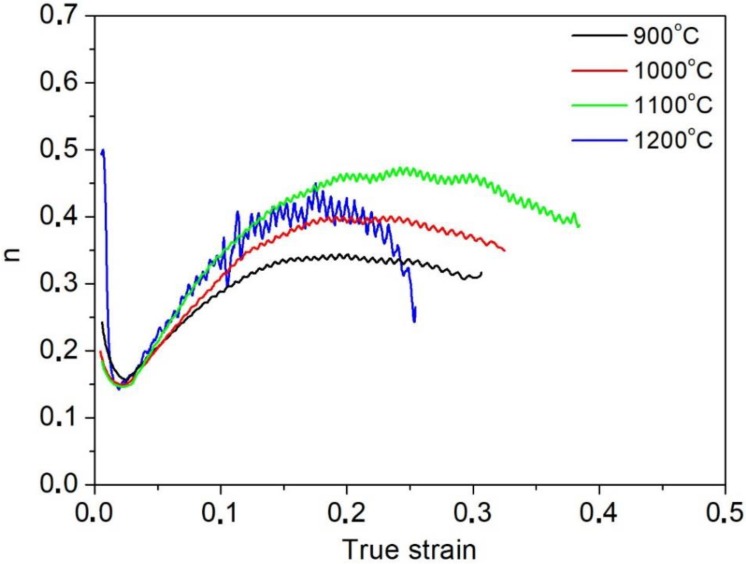
Changes in the work hardening exponent as a function of true strain for the samples treated at different solutioning temperatures.

**Figure 4:**
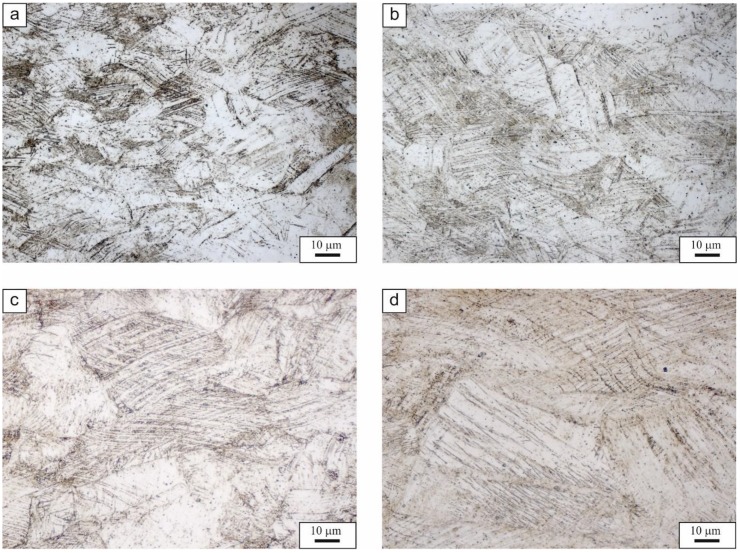
Austenite microstructures with numerous slip bands and twins after tensile tests; the solution annealing temperature: 900 (**a**), 1000 (**b**), 1100 (**c**), and 1200 °C (**d**).

Based on the static tensile tests, values of the work hardening exponent as a function of true strain were also determined for the sample annealed in a temperature range from 900 to 1200 °C (Figure 3). The solution annealing temperature, and thus the corresponding grain size, influences the strengthening process of the tested steel. The highest value of the work hardening exponent n ≈ 0.48 is shown by the sample characterized by the highest elongation (annealing temperature = 1100 °C). The sample solution annealed at this temperature is characterized by the lowest YS_0.2_/UTS ratio value of 0.53, which indicates the high susceptibility to deformation strengthening. At this relatively large grain size, deformation twins are easier to form, which inhibits dislocation movement. The similar tendency is at 1200 °C but only to the strain of ca. 0.2. However, after reaching this strain, necking begins in the sample due to the huge grain size. The surface oxidation taking place in this high temperature is also a real industrial problem and this can affect the mechanical properties to some extent. The corresponding microstructures after the tensile tests with evidences of slip and twins are shown in Figure 4. The strengthening effect is less pronounced in the samples of smaller grain sizes treated at 900 and 1000 °C (Figure 3).

**Figure 5:**
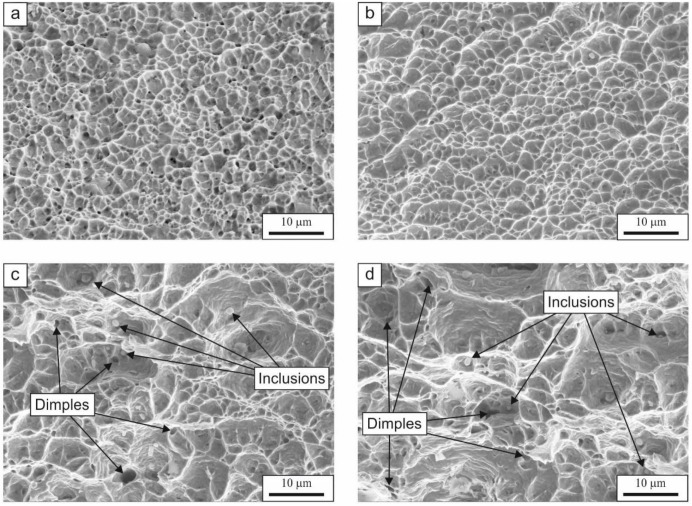
Fracture surfaces of the steel samples after tensile tests treated at different solution annealing temperatures: (**a**) 900, (**b**) 1000, (**c**) 1100, (**d**) 1200 °C.

The tests performed using the scanning electron microscope allowed us to determine the effect of solution annealing temperature on the morphology of fracture surfaces after the static tensile tests. The fractography of the samples is shown in Figure 5. The observations showed that the fractures of the examined steel—regardless of the solution annealing temperature—are ductile, with numerous craters and dimples of varying sizes. The size of the dimples increases considerably at temperatures higher than 1000 °C. The limited elongation of the sample treated at 1200 °C could be attributed to huge differences in the dimple size, which can determine different plastic flow conditions in the microregions. Moreover, macroscopic voids and non-metallic inclusions play important roles in the fracture mechanism [27], especially at the highest temperature, where some C microsegregation effects could appear due to the enhanced diffusion rate [28].

**Figure 6:**
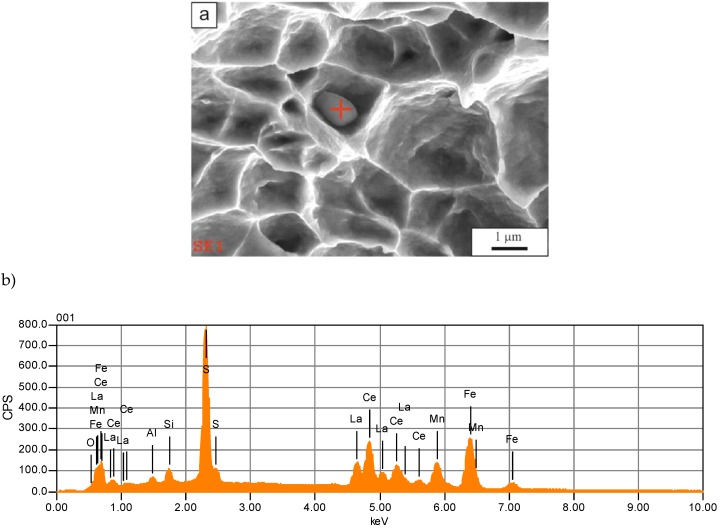
Manganese sulphide partially modified with rare earth elements: (**a**) the inclusion view, (**b**) the EDS spectrum of the inclusion; solution annealing temperature of 1100 °C.

**Figure 7:**
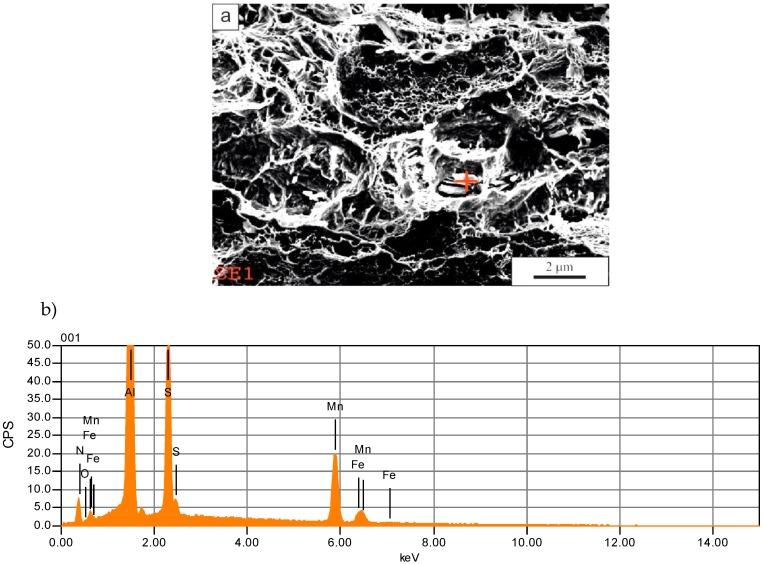
Complex non-metallic inclusion consisting of MnS and AlN: (**a**) the inclusion view, (**b**) the EDS spectrum of the inclusion; solution annealing temperature of 1200 °C.

The fracture surfaces contain small non-metallic inclusions located mainly inside the dimples (Figure 6a). The identified non-metallic inclusions show a globular shape or a near globular shape. In numerous cases, MnS inclusions were identified, which are completely or partially modified with rare earth elements (Figure 6b). Hence, in addition to the spectral lines from S, Mn, O, and Fe, there are also signals from Ce and La on the analyzed spectrograms (mischmetal was added during the melting process). The problem of non-metallic inclusions in high-manganese steels was analyzed in detail in works [28,29,30,31]. It is clear that that there is a relationship between numerous non-metallic inclusions and fracture behavior. The boundaries around the particles are stress concentration areas, which are a reason for microvoids’ formation and their growth during plastic deformation. In addition to the pure or modified sulphides, the complex particles consisting of MnS and AlN are also identified (Figure 7). This was observed earlier by Hongbo et al. [28] in Al-added TWIP steels. They also revealed pure AlN particles or clustered MnS-AlN inclusions, which is consistent with the current study.

## 4. Discussion

The results performed for the 0.054C-24Mn-3.5Si-1.6Al-Nb-Ti high-manganese steel have shown that the varied grain size produced as a result of solution annealing between 900 and 1200 °C determines the mechanical properties considerably. The steels treated at 900 and 1000 °C have fine-grained austenitic microstructures with average grain diameter 13 and 19 μm, respectively. It is possible to refine structure into nano-scale grains, which allows for increasing mechanical properties [19,20,21]. However, the aim of the current study was to investigate the effect of a high-temperature region, which is often applied in real industrial hot-working practice. It was shown that increasing the solution annealing temperature to 1100 ° C results in a distinct grain growth and the formation of heterogeneous, coarse-grained microstructures with a large number of slip lines and twins, which is consistent with the other results in the field [18,19,20]. An increase in the solution annealing temperature in the range 900–1200 °C causes a continuous reduction in the yield stress and tensile strength. The similar behavior was noticed by Lee at al. [32] in Fe-24Mn-4Cr-0.5C steel annealed in the temperature range 800–1100 °C and by Yuan et al. [18] in Fe-25Mn-3Cr-3Al-0.3C-0.01N steel annealed between 700 and 1000°C. The reduction in strength with a simultaneous increase in plasticity corresponding to the increasing grain size is ascribed to the pronounced efficiency of deformation twinning [13,33]. In our case, this is true to the temperature of 1100 °C. Above this temperature, the abnormal grain growth to 225 μm at 1200 °C is a reason for premature neck formation and failure of the sample.

The energy needed to produce twins must be greater than a critical stress. Due to the fact that any factors hindering the dislocation movement cause an increase in critical stress, its value significantly depends on the grain size [34]. The relationship between grain size and critical stress for the formation of deformation twins is usually described by the Hall–Petch equation [35,36,37]:

(2) $$\sigma_{T}=\sigma_{T0}+k_{T}\cdot d^{-1/2}$$

where: $\sigma_{T}$–critical stress necessary for the production of twins, $\sigma_{T0}$–lattice friction stress, $k_{T}$–constant value, *d*–grain size.

According to Equation (2), the critical stress necessary to produce deformation twins decreases with an increasing grain size. In other words, the deformation twins are easier to form in coarse-grained structures. The dislocation activity and the movement of partial dislocations required for the formation of deformation twinning are suppressed by the interaction of relatively high dislocation density in specimens with finer grain sizes, resulting in increasing the twinning stress [32]. The deformation twins act as obstacles for dislocations motion leading to a decrease of the dislocation glide distance. It is related to the decrease of the dislocation mean free path due to progressive fragmentation of the austenitic grains into smaller constituents by newly formed deformation twins. The nucleation of new deformation twins results in an increasing fragmentation of the grains by twins with increasing strain (Figure 4). This result is called the dynamic Hall–Petch effect [13,33], which increases the strain hardening rate. Deformation twins nucleate in the grain boundary region. During the process of deformation twinning, the source of deformation twins generates other dislocations on the opposite side of the grain [38].

The most effective strain hardening behavior and the corresponding balance of strength–ductility was noted for the steel annealed at 1100 °C (Figure 3). The strain hardening exponent in coarse-grained alloys is higher at 1110 and 1200 °C than for the fine-grained alloys (with an annealing temperature range between 900 and 1000 °C). The similar tendency was noted by Sevsek et al. [6] in X30MnAl17-1 steel characterized by various grain sizes. They observed the higher twin density in coarse-grained steels as opposed to the fine-grained specimens.

**Figure 8:**
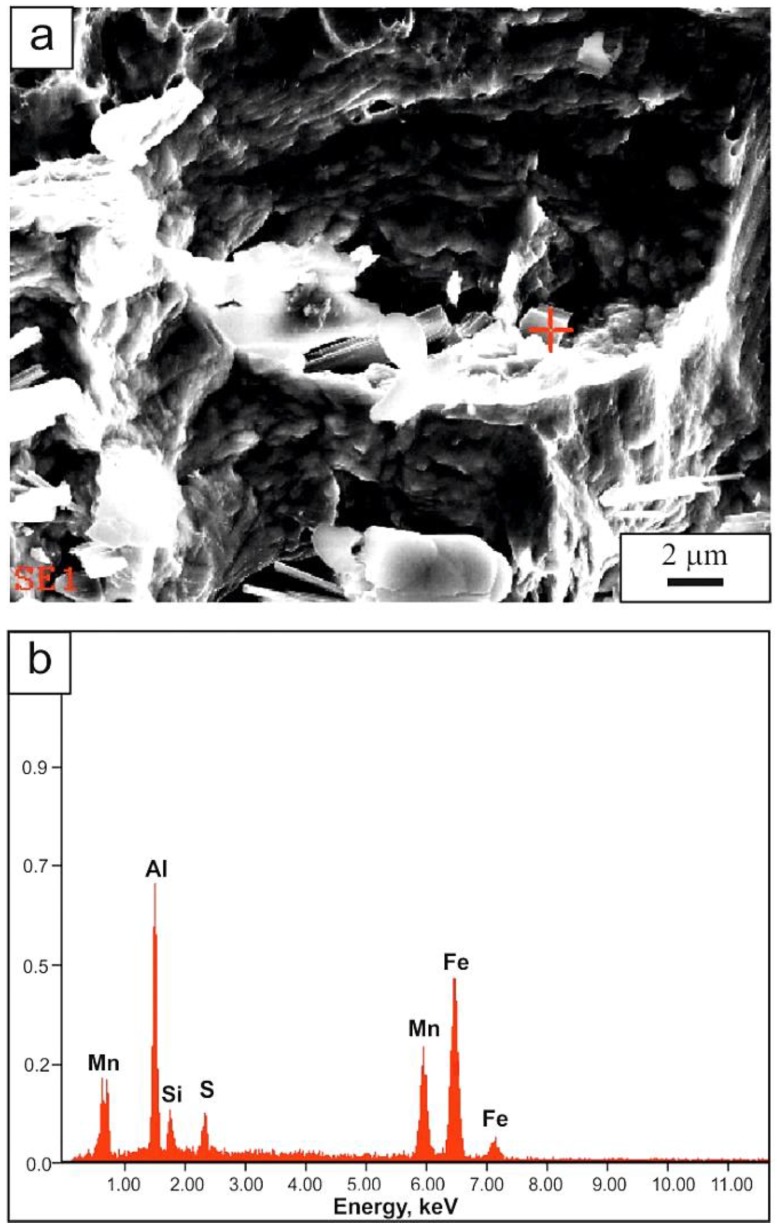
AlN-rich polygonal particles decorating dimples: (**a**) the inclusion view, (**b**) the EDS spectrum of the inclusion; solution annealing temperature of 1200 °C.

A deviation from the ductility trend described above is the result obtained for the sample solution annealed at 1200 °C, showing a slightly different course of the tensile curve (Figure 2) and the increased ratio of YS/UTS = 0.58 (Table 3). The reason can be related to the huge grain size above 200 μm and the heterogeneous dimples observed at the fracture surface (Figure 5d). The macroscopic necking behavior can be of higher importance under such deformation conditions. It means that the acceleration of void formation during post-necking decohesion is more important than the interaction between dislocations and twins in a microscale. The crucial role of the fracture behavior is based on the inclusion of MnS (Figure 6), complex non-metallic inclusions containing MnS and AlN (Figure 7), and single polygonal AlN particles. Figure 8 shows the polygonal AlN particles decorating dimples in the steel annealed at 1200 °C.

**Figure 9:**
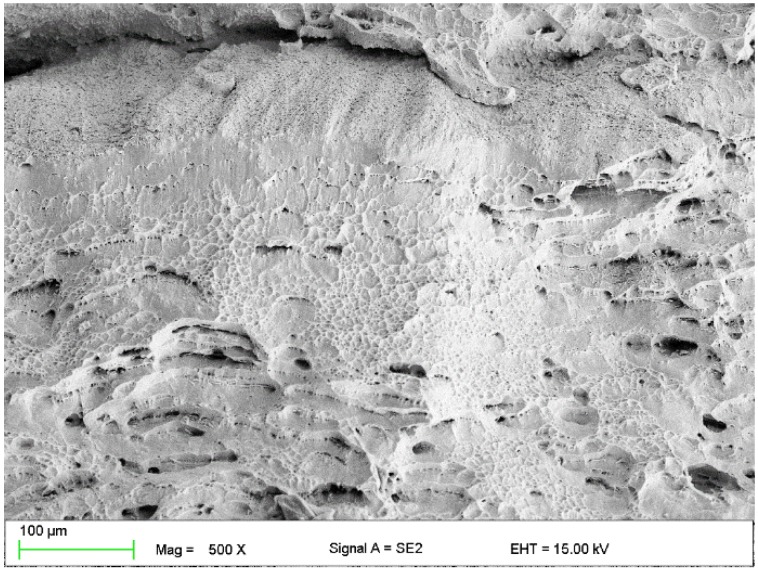
The fracture surface of the sample annealed at 1200 °C, showing interactions between the formed macrovoids and numerous non-metallic inclusions.

The number of such inclusions in TWIP steels is high due to the high Mn addition, which is further increased in Al-added high-Mn steels. Yang et al. [27] reported that the non-metallic inclusions are the most common initiation sites for macroscopic voids. The small voids formed around the non-metallic inclusions will coalescence due to the increasing strain and corresponding stress concentration. Such interactions between the non-metallic inclusions and macroscopic voids can be seen in the fracture surface of the specimen annealed at 1200 °C (Figure 9). Moreover, Yang et al. [27] observed that due to numerous non-metallic inclusions in high-manganese steels, the voids can be formed even before necking, which it is not typical for ductile materials. This can explain the premature decrease in the work hardening exponent in Figure 3 and is consistent with the results obtained in [27]. This premature necking behavior and subsequent low ductility were attributed to the multiplication of macroscopic voids during plastic deformation.

## 5. Conclusions

The effect of different grain sizes on the microstructure and strain hardening behavior of low-C high-Mn steel solution heat treated in a range from 900 to 1200 °C was analyzed. The clear relationship between the grain size of the austenite and the mechanical properties was found in a temperature range between 900 and 1100 °C. The strength properties increase considerably with a decrease in grain size from 225 to 13 μm, wherein the critical grain growth took place between 1000 and 1100 °C. The inverse relationship was confirmed between the grain size and ductility. The uniform elongation was decreasing from 48% to 39% along with the reduction in grain size. The samples were characterized by a typical ductile type of fracture, with the dimples’ size corresponding to the grain size. The crucial role of pure MnS, modified MnS, complex MnS+AlN, and pure AlN particles in intense macrovoid formation and propagation during straining was revealed. The premature fracture and lowest ductility of the specimen annealed at 1200 °C is due to the complex interaction of abnormal grain growth, non-metallic inclusions, and macrovoid coalescence and growth, which dominated over the interactions between dislocations and twins following the dynamic Hall–Petch relationship.

## Figures and Tables

**Table 1:** Chemical composition of analyzed steel.

C	Mn	P	S	Si	Al	Nb	Ti	N	O
0.054	24.4	0.004	0.016	3.5	1.6	0.029	0.075	0.0039	0.0006

**Table 2:** Effect of the solution heat-treated temperature on grain size.

Solution Heat-Treatment Temperature, °C	Average Diameter, μm	Average Grain Area, μm^2^	Mean Intercept, μm	Grain Size No. G	Standard Deviation of the Diameter, μm
900	13.0	173	11.6	9.5	4.8
1000	18.5	346	16.4	8.5	6.9
1100	136.6	16,973	121.0	3.0	25.4
1200	225.5	47,357	200.7	1.5	38.7

**Table 3:** Effect of grain size on mechanical properties.

Solution Heat-Treatment Temperature, °C	Average Diameter, μm	YS_0.2_ * MPa	UTS * MPa	YS_0.2_/ UTS	UEl * %	RA * %
900	13.0	480 ± 10	769 ± 7	0.62	39.3 ± 3.0	42.0 ± 1.5
1000	18.5	443 ± 15	740 ± 17	0.59	42.1 ± 2.5	45.2 ± 0.8
1100	136.6	335 ± 12	630 ± 12	0.53	48.0 ± 2.2	51.0 ± 2.0
1200	225.5	314 ± 16	539 ± 20	0.58	29.9 ± 3.5	39.5 ± 3.1

* The average value calculated from three measurements.
